# 60-GHz Millimeter-wave Over Fiber with Directly Modulated Dual-mode Laser Diode

**DOI:** 10.1038/srep27919

**Published:** 2016-06-14

**Authors:** Cheng-Ting Tsai, Chi-Hsiang Lin, Chun-Ting Lin, Yu-Chieh Chi, Gong-Ru Lin

**Affiliations:** 1 Graduate Institute of Photonics and Optoelectronics, National Taiwan University (NTU), No. 1, Sec. 4, Roosevelt Rd., Taipei 10617, Taiwan, Republic of China; 2 Institute of Photonic System, National Chiao Tung University (NCTU), No. 301, Gaofa 3rd Rd., Guiren Dist., Tainan City 71150, Taiwan, Republic of China

## Abstract

A directly modulated dual-mode laser diode (DMLD) with third-order intermodulation distortion (IMD_3_) suppression is proposed for a 60-GHz millimeter-wave over fiber (MMWoF) architecture, enabling new fiber-wireless communication access to cover 4-km single-mode-fiber (SMF) and 3-m wireless 16-QAM OFDM transmissions. By dual-mode injection-locking, the throughput degradation of the DMLD is mitigated with saturation effect to reduce its threshold, IMD_3_ power and relative intensity noise to 7.7 mA, −85 dBm and −110.4 dBc/Hz, respectively, providing huge spurious-free dynamic range of 85.8 dB/Hz^2/3^. This operation suppresses the noise floor of the DMLD carried QPSK-OFDM spectrum by 5 dB. The optical receiving power is optimized to restrict the power fading effect for improving the bit error rate to 1.9 × 10^−3 ^and the receiving power penalty to 1.1 dB. Such DMLD based hybrid architecture for 60-GHz MMW fiber-wireless access can directly cover the current optical and wireless networks for next-generation indoor and short-reach mobile communications.

Nowadays, the 60-GHz millimeter wave (MMW) carrier with high bandwidth availability and license-free features has comprehensively investigated to develop next-generation indoor wireless networks, which effectively releases the explosive data traffic with the use of carriers at ultra-high frequency (UHF) and super-high frequency (SHF) bands^1^. As the 60-GHz MMW carrier exhibits an inherently limited transmission distance due to high atmospheric attenuation^2^, it is mainly utilized to construct high-speed communication links for local wireless access applications such as home area networks (HAN) and wireless personal area networks (WPAN)^3^,^4^. More recently, the optical fiber and MMW combined wireless access architecture (also called MMW-over-fiber (MMWoF) system) has also emerged as the promising solution for extending the service range and fusion capability of the 60-GHz MMW wireless communication^5^,^6^,^7^,^8^. Architecturally, such a system delivers baseband data with an optical carrier over fiber^9^,^10^,^11^,^12^, which then converts its central carrier frequency up to MMW bands at an optical receiving end by using an electrical mixer and a remote MMW local-oscillator; however, such a design is difficult to be realized for practical applications as the necessity of cost-ineffective MMW devices added at the user end. To simplify the fiber-wireless access architecture, the optical self-beating method is thus employed at receiving part, which mutually beats the dual-mode light source (DMLS) at the photodetector (PD) for all-optical generation of MMW carrier^13^,^14^,^15^. Figure 1 illustrates the architecture of 60-GHz MMWoF system which provides fiber-wireless access network for indoor wireless communication based on the use of DMLS as a MMW embedded optical carrier.

Typically, the compact DMLS is approached by coupling two distributed feedback laser diodes (DFBLDs); however, the phase difference between two individually lasing DFBLDs usually makes the beat MMW carrier unstable with residual frequency sweeping phenomenon, which needs an additional microwave phase-locked loop to synchronize two free-running DFBLDs^16^. Later on, a central-carrier suppressed double-sideband source has emerged to form a coherent DMLS by seeding a continuous-wave (CW) single-mode light into a RF modulated and nully biased Mach-Zehnder modulator (MZM)^17^,^18^. By further using a dual-MZM consisted I/Q modulator, the double-sideband carriers can construct a 24-Gb/s data link over 40-km single-mode-fiber (SMF) and 1.5-m wireless transmissions^19^. As the SMF transmission introduced chromatic dispersion can lead to the phase difference between the data carried by upper and lower sideband carriers, it still suffers from the power fading effect after the self-beating at receiving part^20^. Although the optical single-sideband modulation can be employed to overcome the fiber dispersion^21^, it demands the use of higher-speed MZM and synthesizer.

Recently, a directly modulated dual-mode injection-locking laser source was employed to eliminate the need for an external modulator^22^. In view of previous works, the dual-mode master source employs the aforementioned dual-DFBLD architecture, which concurrently injection-locks two longitudinal modes in a Fabry-Perot laser diode (FPLD) to realize the DMLS^23^. Similarly, the wavelength fluctuation between these DFBLDs inevitably perturbs the frequency of the DMLS beat MMW carrier. Indeed, the double-sideband light source with high coherence can be regarded as a potential master for performing the dual-mode injection-locking, but the injection efficiency is still limited by the serious competition between lasing modes of the slave FPLD. After reducing the front-facet reflectance of the conventional FPLD, a colorless FPLD with TO-can package has been constructed as a qualified slave laser with improved dual-mode injection-locking efficiency^24^,^25^. To effectively use the limited bandwidth of the To-can packaged colorless FPLD, a high spectral-efficiency data format which combines quadrature amplitude modulation (QAM) with orthogonal frequency division multiplexing (OFDM) was introduced^26^. After implementing the dual-mode injection-locking with double-sideband master, such a colorless FPLD can deliver the 36-Gb/s 16-QAM OFDM data over 25-km SMF and perform the MMW wireless transmission at 4 Gb/s^27^. Essentially, the dual-mode injection-locking promotes the gain competition of chosen longitudinal modes in the slave laser, which reduces the relative intensity noise (RIN) induced by non-coherent spontaneous emission such that the modulation bandwidth of the slave laser can be greatly enlarged^28^. When considering the direct modulation, the injection-locking can provide a large amount of coherent photons to further suppress the nonlinear distortion of the slave laser^29^,^30^.

In this work, with the use of a third-order intermodulation distortion (IMD_3_) suppressed dual-mode laser diode (DMLD), a 60-GHz MMW embedded fiber-wireless access architecture is demonstrated for delivering the 6-Gb/s 16-QAM OFDM data over 4-km SMF and 3-m wireless transmissions. After dual-mode injection-locking with a double-sideband master, the declined frequency response and the reduced RIN of the DMLD at different injection powers are characterized. The suppressed nonlinear distortion is observed by employing the two-tone modulation analysis to examine the spectrum of the DMLD carried QPSK-OFDM data. The power of the carried QPSK-OFDM data is optimized after detuning the injection power of the DMLD. By analyzing the complementary cumulative distribution functions (CCDFs) of the peak-to-average power ratio (PAPR), the RIN is suppressed to minimize the QAM-OFDM waveform distortion. In addition, the SMF transmission introduced power fading effect on the DMLD carried QAM-OFDM data is also numerically simulated to examine its influence during transmission. By employing the 16-QAM OFDM data, the transmission performances including signal-to-noise ratio (SNR), error vector magnitude (EVM) and bit error rate (BER) are examined at different optical receiving powers after 60-GHz wireless transmission over 3 m on free space. Finally, the temperature related optical stability of the dual-mode injection-locked DMLD is also discussed.

## Results

### Contribution of the dual-mode injection-locking to the transmission performance of the slave laser

For dual-mode operation, the adjacent dual longitudinal modes of a LD with 600-μm cavity length of and 2% front-facet reflectance is injection-locked by a dual-sideband modulated single-mode master with a central carrier suppression ratio (CCSR) of 15.5 dB. Although the CCSR of the dual-sideband master is sufficiently high, the residual central carrier still exists to induce four-wave-mixing (FWM) components that would seriously degrade the long distant transmission by suffering from the chromatic dispersion.

**Figure 2:**
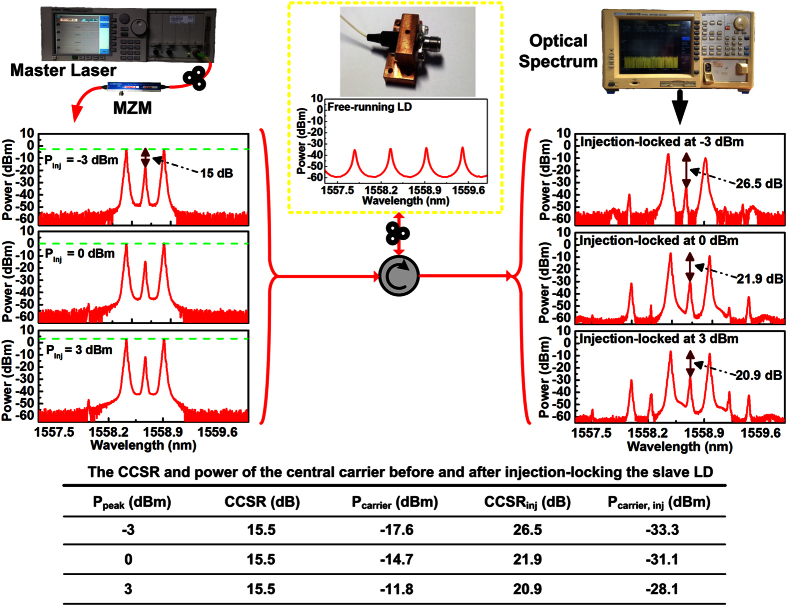
The optical spectra, related powers and CCSRs of the dual-sideband master and injection-locked slave LD at different injection powers. As the central carrier of the dual-sideband master deviates from the resonant wavelength of the slave LD, it can be significantly suppressed after injection-locking.

Fortunately, the injection-locking plays an important role to further suppress the central carrier coming from the dual-sideband master as it is not located at the wavelength of any resonant longitudinal mode of the slave LD. With the dual-mode injection-locking, the slave LD not only provides the resonant dual-mode output for remotely self-beating MMW carrier, but also suppresses the residual central carrier component from the dual-sideband master. Figure 2 shows the optical spectra of the dual-sideband master and the dual-mode injection-locked slave. By amplifying the peak power of the dual-sideband master from −3 to 3 dBm, the CCSR is still fixed at 15.5 dB, but the power of the residual carrier component distinctly increases from −17.6 to −11.8 dBm. As such a central carrier deviates from the resonant wavelength of the slave LD, it is suppressed after injection-locking although it still obtains less gain due to the weak resonance of the slave LD. This contributes to decrease the spontaneous emission and reduce the RIN of the slave LD. To compare, the CCSR and power of the central carrier before and after injection-locking the slave LD are summarized in Fig. 2. Note that although the CCSR can be improved after injection-locking, it is somewhat reduced if further enlarging the peak power of the dual-sideband master.

**Figure 3:**
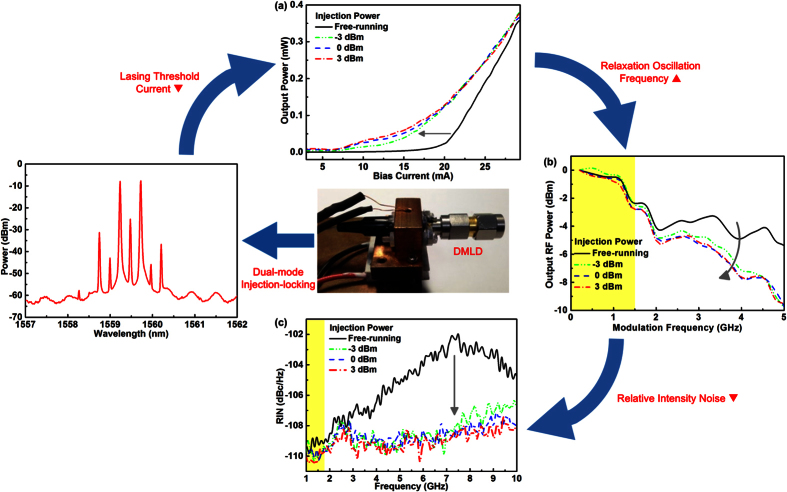
The output characteristics of the free-running and injection-locked slave LD. (**a**) The power-to-current plot, (**b**) the modulation response and (**c**) the RIN spectrum of the slave LD reveal that the threshold reduction, throughput declination and RIN suppression are reduced after dual-mode injection-locking.

With dual-mode injection, the power-to-current responses of the slave LD are shown in Fig. 3(a). After dual-mode injection-locking with a peak power of −3 dBm, the slave LD greatly reduces its lasing threshold current from 20 to 8.8 mA, which enables the slave LD to handle larger OFDM power without suffering from waveform clipping induced data distortion under same bias. In comparison with the single-mode injection-locking case, the dual-mode master provides a large number of coherent photons to enhance the stimulated emission in the slave LD; however, it becomes easier to saturate the slave LD under the same operation. Hence, enlarging the peak injection power from −3 to 3 dBm only reduces the lasing threshold from 8.8 to 7.7 mA. Not only the threshold current but also the modulation throughput of the slave LD can be affected by the dual-mode injection-locking.

Figure 3(b) shows that the frequency response of the free-running LD is declined after the dual-mode injection-locking at power of −3 dBm. Fortunately, the throughput power only decreases from −2.28 to −2.58 dBm at modulation within 1.5 GHz that is used for carrying the OFDM data, as indicated by the colored area shown in Fig. 3(b). The throughput power declination is always observed due to the frequency roll-off effect occurred in the injection-locked laser diode^31^,^32^,^33^, which degrades the transmission performance of the carried OFDM data^24^. Note that the modulation throughput of the DMLD rapidly declines as the power at low frequencies is converted to high frequencies by shifting up its relaxation oscillation frequency. When increasing the injection power from −3 to 3 dBm, the throughput power of the DMLD at 1.5 GHz further reduces from −2.58 to −2.76 dBm and reveals a saturation trend due to strong injection. To optimize the SNR performance, Fig. 3(c) shows that the RIN level of the slave LD can be reduced with dual-mode injection-locking. Injection-locking at −3 dBm shifts up the RIN peak frequency of the slave LD from 7.25 to 10 GHz and suppresses it from −102 to −107.5 dBc/Hz. This contributes to slightly reduce the noise level of the RIN spectrum at 1.5 GHz by 0.8 dBc/Hz. Enlarging the injection power from −3 to 3 dBm further suppresses the RIN at 1.5 GHz to −110.4 dBc/Hz, revealing that the low-frequency RIN can still be suppressed even under saturated injection-locking condition. Although the injection-locking declines the throughput slope of the slave LD, which can be saturated with sufficient injection to reduce the intensity noise for improving the SNR of carried OFDM data.

### Suppression of the nonlinear modulation distortion in the LD under dual-mode injection-locking

**Figure 4:**
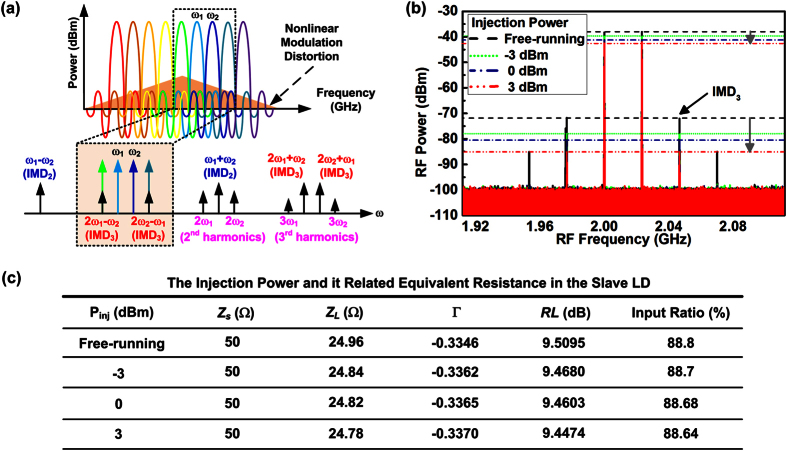
The DMLD carried OFDM data and its related intermodulation distortion terms. (**a**) The DMLD induced third-order intermodulation distortion (IMD_3_) spectral pedestals after modulating the QAM-OFDM data. (**b**) The frequency spectra of the DMLD carried two-tone RF signal at different injecting powers. (**c**) The equivalent resistance and related input ratio in the DMLD at different injecting powers. The IMD_3_ can be suppressed by enlarging the injection power.

Under large-signal modulation, the DMLD induced nonlinear modulation distortion always occurs to degrade the transmission performance of the carried OFDM data. Figure 4(a) illustrates the schematic spectrum of the DMLD carried OFDM data. When considering two adjacent OFDM subcarriers (ω_1_ and ω_2_), the IMD_3_ generates unwanted spectral peaks at 2ω_1 _± ω_2_ and 2ω_2 _± ω_1_, and the low frequency terms and its carried data overlap with the nearby OFDM subcarriers (2ω_1_ − ω_2_ and 2ω_2_ − ω_1_) to induce signal crosstalk and SNR degradation. As the adjacent OFDM subcarriers beat each other to cause related IMD_3_ peaks, the multi-OFDM carrier modulation inevitably induces the nonlinear modulation distortion with numerous IMD_3_ components within the modulation bandwidth to result in a broadband intensity noise (see the colored area of Fig. 4(a)).

When modulating the DMLD by the two-tone RF signal at a peak power of 6 dBm, the induced IMD_3_ spectral peaks are shown in Fig. 4(b). The magnitude of these IMD_3_ spectral components associated with the linearly modulated spectra can be treated as a figure-of-merit for evaluating the performance of the carried OFDM data, because it is near the fundamental components of the modulated signal and located within the modulation bandwidth. In principle, the power of the two-tone RF signal seeded into the DMLD is related to the equivalent impedance (*Z*_*L*_) of the slave LD which dominants the return loss (*RL*) before direct modulation. The *RL* can be expressed as *RL* = *−20*  log_*10*_|Γ| in unit of dB, where Γ denotes the reflection coefficient given by Γ  =  *Z*_*L*_ − *Z*_*S*_*/Z*_*L*_ + *Z*_*S*_ with *Z*_*S*_defining the impedance of the RF signal source. The *RL* is also expressed as *RL* = *10*  log_*10*_(*P*_*i*_*/P*_*r*_) with *P*_*i*_ and *P*_*r*_ denoting powers of input and reflection, which can be used to calculate the reflection ratio (*P*_*i*_*/P*_*r*_) of the two-tone RF signal by substituting the *P*_*i*_ at 1 mW.


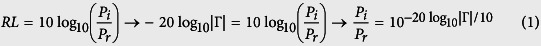


As the dual-mode injection-locking provides numerous coherent photons to enhance stimulated emission and release lasing threshold of the slave LD, the required driving current at fixed bias point effectively decreases to reduce the *Z*_*L*_ of the slave LD, as summarized in Fig. 4(c). By substituting the *Z*_*S*_with 50 Ω into aforementioned formula, the *RL* of 9.51 dB can be obtained for the free-running slave LD, which is reduced from 9.47 dB to 9.45 dB after injection-locking the DMLD with a power enlarged from −3 dBm to 3 dBm. Considering the RF signal with a power of 1 mW, its input ratio that obtains with calculating the reflection ratio is only decreased from 88.7% to 88.6% when increasing the injection power of the two-tone modulated DMLD from −3 to 3 dBm.

In principle, the amplitude of the IMD_3_ is proportional to the third order of the optical modulation index given by *m* = *P*_*signal*_*/*<*P*> with *P*_*signal*_ denoting the amplitude of modulated signal and <*P*> the average power of the DMLD^34^,^35^,^36^. Once the input ratio of the *P*_*signal*_ can be decreased by enlarging the injection power of the DMLD from −3 to 3 dBm, the reduced *P*_*signal*_ can contribute a suppression of the IMD_3_ terms. Since the IMD_3_ of the slave LD can be suppressed by employing the injection-locking technology^29^,^30^, the peak power of the IMD_3_ terms can be suppressed from −72 to −78 dBm under dual-mode injection-locking at power of −3 dBm. In addition, not only the throughput power of the DMLD but also the input ratio of the carried two-tone RF signal can be reduced after the injection-locking. This degrades the carried two-tone RF signal power so as to provide the IMD_3_ peak suppression, as the IMD_3_ spectral peak power is proportional to third order of the optical modulation index. The increase of the injection power from −3 to 3 dBm further suppresses the IMD_3_ peak power from −78 to −85 dBm, revealing that the nonlinear modulation distortion in the DMLD is greatly reduced under strong injection-locking.

**Figure 5:**
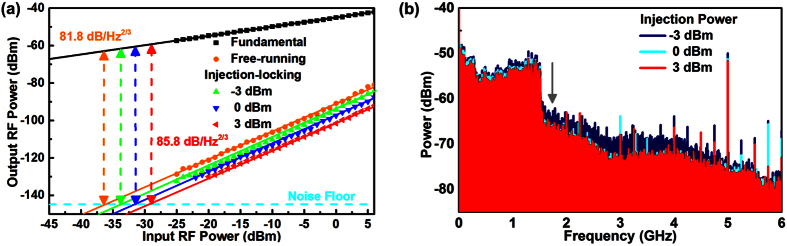
The improvement on the transmission performance of the IMD_3_ suppressed DMLD. (**a**) The spurious-free dynamic ranges (SFDRs) of the injection-locked LD at different injection powers. As the IMD_3_ peaks of the DMLD is greatly suppressed by injecting up to 3 dBm, it enlarges the related SFDR for carrying the QPSK-OFDM data. (**b**) The RF spectrum of the DMLD carried QPSK-OFDM data after optical BtB and 3-m wireless transmissions, in which the improved SFDR of the DMLD effectively reduces the noise level of the carried QPSK-OFDM spectrum.

To further investigate the IMD_3_ suppression in detail, the spurious-free dynamic range (SFDR) is employed as the figure of merit for obtaining the tolerance of modulating RF power. The RF spectrum of the DMLD carried two-tone RF signal is measured by setting the resolution bandwidth (RBW) of the spectrum analyzer down to 1 Hz, as shown in Fig. 5(a). Note that a SFDR as high as 81.8 dB/Hz^2/3^ is observed for the free-running slave DMLD (with comparing the systematic background noise floor of −145 dBm/Hz), and it could be further improved from 83.1 to 85.8 dB/Hz^2/3^ by dual-mode injection-locking the slave DMLD at an optical power enlarged from −3 to 3 dBm, which is treated as a direct evidence for the IMD3 suppression.

Afterwards, the suppression on the nonlinear modulation distortion is also surveyed when modulating the DMLD with a 3-Gb/s QPSK-OFDM data. After experiencing the optical back-to-back (BtB) and 3-m wireless transmissions, the spectra of the DMLD carried QPSK-OFDM data at a power level of 6 dBm are illustrated in Fig. 5(b). Since the nonlinear modulation distortion introduces a large amount of IMD_3_ spectral peaks in the DMLD, the modulated OFDM data spectrum exhibits a broadband nonlinear noise level ranged from DC to 5 GHz. Increasing the injection-locking power from −3 to 3 dBm not only suppresses the IMD_3_ peaks but also reduces the RIN and the noise floor of the OFDM spectrum by 5 dB (from −60 to −65 dBm at a frequency of 1.5 GHz). Apparently, the suppression on nonlinear modulation distortion can be observed by dual-mode injection-locking the DMLD, which effectively reduces the IMD_3_ spectral peaks and improves the OFDM data transmission performance. Although the dual-mode injection degrades the throughput power and input ratio of the DMLD, which concurrently contributes to suppress the IMD_3_ spectral peaks.

### Optimizing the transmission BER by adjusting the DMLD bias and the power of carried OFDM data

**Figure 6:**
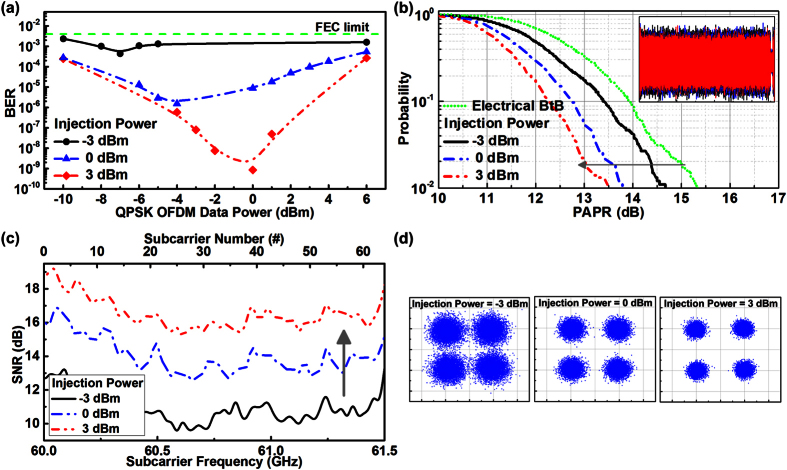
The transmission performance of the DMLD carried QPSK-OFDM data. The (**a**) BER, (**b**) CCDF, (**c**) SNR and (**d**) constellation plots of the DMLD carried QPSK-OFDM data at different injection powers. By optimizing the BER and CCDF of the DMLD carried QPSK-OFDM data, its related SNR can be effectively enlarged after optical BtB and 3-m wireless transmissions.

To optimize the transmission performance of the DMLD carried QPSK-OFDM data, the BER responses at different data and injection-locking powers are analyzed and shown in Fig. 6(a). When injection-locking the DMLD at −3 dBm under a bias current of 50 mA, the BER of the carried OFDM data can be reduced from 2.3 × 10^−3^ to 4.3 × 10^−4^ by enlarging the data power from −10 to −7 dBm, indicating that the modulation depth as well as the on/off extinction of the carried OFDM data is increased currently. However, the continually enlarged data power from −6 to 6 dBm inversely degrades the BER from 4.3 × 10^−4^ to 2.3 × 10^−3^ as the OFDM waveform clipping induces accordingly. Although the BER of the OFDM data carried by the DMLD under an injection of −3 dBm remains below the FEC criterion of 3.8 × 10^−3^, the BER performance can still be slightly improved with enlarged injection level, as the threshold current and the RIN of DMLD can be suppressed to mitigate the waveform clipping and intensity fluctuation occurred on the OFDM waveform. In this way, the optimized OFDM data power can be scaled up from −7 to 0 dBm to provide a lowest BER of 8.5 × 10^−10^ when enlarging the injection power from −3 to 3 dBm. Figure 6(b) illustrates the CCDFs of the PAPR for the OFDM data carried by the DMLD injection-locked at different powers. Note that the carried OFDM data power is optimized at each injection power in this experiment. As the RIN induces high-frequency noise in the DMLD, it introduces unwanted noise signal in time-domain that instantaneously enlarges the OFDM waveform peak and increases the related PAPR after superposition. Increasing the injection level from −3 to 3 dBm shifts up the frequency and suppresses down the power of the RIN peak of the DMLD to reduce the PAPR of the carried OFDM data from 14.6 to 13.5 dB at the specified probability of 10^−2^.

As expected, the inset of Fig. 6(b) shows the less fluctuated OFDM waveform in time-domain with suppressing the RIN in frequency domain. In particular, as the optimized modulation depth is obtained by maximizing the on/off extinction of the OFDM data, such an operation may cause a slight waveform clipping so that the PAPR of the DMLD carried OFDM waveform is always smaller than that of the electrical BtB transmitted OFDM waveform. Figure 6(c,d) illustrate the SNR spectra and constellation plots of the DMLD carried QPSK-OFDM data after the BtB fiber wired and 3-m wireless transmissions, respectively. At the injection power of −3 dBm and the data power of −9 dBm, the average SNR and EVM of the carried OFDM data is 10.8 dB and 28.8%, respectively, which meets the demands of the FEC criterion of 8.5 dB and 37% (at the BER of 3.8 × 10^−3^). When enlarging the injection and the data power up to 3 and 0 dBm, respectively, not only the nonlinear distortion and the RIN of the DMLD can be suppressed, but also the waveform clipping in the OFDM waveform can be mitigated, which effectively improves the average SNR and EVM of the carried OFDM data to 16.5 dB and 14.9%, respectively.

### The SNR and BER performances of the DMLD carried QAM-OFDM data after the wired and wireless transmissions

To enrich different access services of the 60-GHz fiber-wireless access architecture, the DMLD carried OFDM data can either be received as optical baseband or MMW wireless data; however, the receiving power of SMF transmitted OFDM data is inherently degraded by the dispersion induced power fading effect. Without considering the attenuation in SMF, the receiving power of each OFDM subcarrier after SMF transmission can be expressed as^37^ *P*_*r*_ = (*1* + α)cos^*2*^(*2*π^*2*^*β*_*2*_*Lf*^* 2*^ − tan^*−1*^α) × *P*_*OFDM*_, where *β*_*2*_ denotes the group velocity dispersion of the SMF (*β*_*2*_ = −22.11 ps^2^/km), *L* the fiber length, α the linewidth enhancement factor, *f* the OFDM subcarrier frequency, *P*_*OFDM*_ the receiving power of the OFDM data without power fading. At a certain frequency, the *P*_*r*_ becomes zero when the term |*2*π^*2*^*β*_*2*_*Lf*^*2*^ − tan^*−1*^α| approaches π*/2*. As the group delay can be enlarged by increasing the SMF length, the power fading induced notch can be down-shifted to low frequencies such that the SNR performance of the received OFDM data is degraded accordingly. By substituting the carrier-induced differential refractive index of DMLD with −1.8 × 10^−26 ^m^3^ into aforementioned formula^38^, the α of 1.12 can thus be obtained to perfectly simulate the related power fading^39^,^40^.

**Figure 7:**
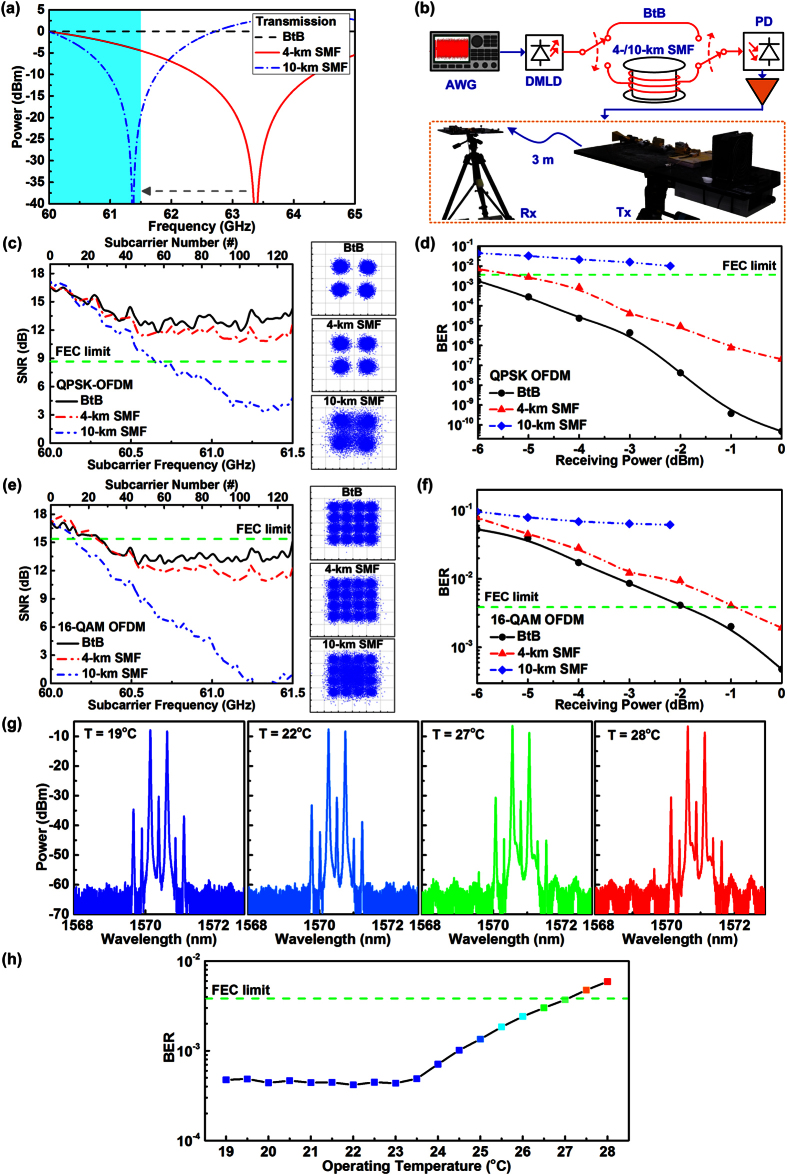
The power fading and the transmission performance of the DMLD carried QPSK-/16-QAM OFDM data after 4-/10-km SMF and 3-m free-space propagations. (**a**) The receiving power response of the DMLD after SMF transmission with different lengths. (**b**) The testing bench for measuring the transmission performance of the DMLD carried QAM-OFDM data. By lengthening the SMF, the power fading induced spectral notch down-shifts to seriously degrade the receiving power of the DMLD carried OFDM data. (**c**) The SNR response and constellation plots of the DMLD carried QPSK-OFDM data. (**d**) The BER response of the DMLD carried QPSK-OFDM data at different optical receiving powers. (**e**) The SNR response and constellation plots of the DMLD carried 16-QAM OFDM data. (**f**) The BER response of the DMLD carried 16-QAM OFDM data at different optical receiving powers. With sufficient optical receiving power, the 6 Gb/s 16-QAM OFDM data can be delivered by the DMLD based fiber-wireless access architecture after 4-km SMF and 3-m wireless transmissions. (**g**) The optical spectra of the injection-locked DMLD at different operating temperatures. (**h**) The BER performances of the DMLD carried 16-QAM OFDM data at different operating temperatures.

As shown in Fig. 7(a), the power fading induced notch is located at 63.5 GHz after passing through a 4-km SMF, which is down-shifted to 61.4 GHz by lengthening the SMF length to 10 km. As the down-shifted notch has already entered the preset modulation bandwidth of the OFDM data (colored area in Fig. 7(a)), which seriously attenuates the data receiving power so as to degrade the corresponding SNR. To observe and verify the power fading induced SNR degradation in experiments, both SNR spectrum and constellation plot of optical baseband and MMW wireless transmitted QPSK-OFDM data at optical receiving power of −3 dBm are analyzed after 4-/10-km SMF and 3-m free-space propagations, as illustrated in Fig. 7(b). Under optical BtB transmission without SMF, the average SNR of 3-m wireless transmitted OFDM data carried by MMW is up to 13.9 dB without the influence of power fading effect, which reveals a clear constellation plot with an EVM of 20%, as shown in Fig. 7(c). If the optical path lengthens with adding 4-km SMF, the SNR of wireless transmitted OFDM data at 61.5 GHz slightly decreases from 13.9 to 13.5 dB due to the additive power fading, which concurrently degrades its EVM from 20% to 21%. Moreover, the power fading induced notch could inevitably enter the modulation bandwidth of carried OFDM data (located at 61.36 GHz) to seriously degrade its average SNR to 10.9 dB when lengthening the SMF to 10 km. Figure 7(d) illustrates the BER responses of the DMLD carried QPSK-OFDM data at different optical receiving powers. Under optical BtB transmission, the lowest BER of 4.6 × 10^−11^ can be obtained at an optical receiving power of 0 dBm for the 3-m wireless transmitted OFDM data.

After lengthening the optical path with 4-km SMF, the lowest BER of received OFDM significantly degrades from 4.6 × 10^−11^ to 2 × 10^−7^ due to the induced MMW power fading, which reveals a receiving power penalty of about 1 dB. When considering optical baseband transmission in 10-km SMF, because the enlarged attenuation and aggravated power fading seriously degrades the SNR of the received OFDM data, all BER responses cannot meet the FEC criterion as the receiving power penalty concurrently increases to >3.1 dB. To enlarge the transmission capacity of our fiber-wireless access architecture, the modulation level of the OFDM data-stream is increased from QPSK to 16-QAM. Figure 7(e) shows the SNR spectra and the constellation plots of the 3-m wireless transmitted 16-QAM OFDM data at optical receiving power of −3 dBm. Under optical BtB transmission, the average SNR and the EVM of the wireless transmitted OFDM data is 14.4 dB and 19%, respectively, which is fail to meet the 16-QAM OFDM required FEC criterion of 15.2 dB and 17.3%.

As the 16-QAM OFDM is more complicated and sensitive than QPSK-OFDM, its performance is easier to be effected by the SMF transmission induced power fading. By extending the optical path by 4-/10-km SMF, the received SNR and EVM of 3-m wireless transmitted OFDM data is degraded to 14.2 and 9.3 dB and to 19.3% and 34.4%, respectively. Although the MMW power fading effect is severely inferenced the SNR performance of DMLD carried 16-QAM OFDM data, there is still an opportunity to meet the FEC demand by simply enlarging the optical receiving power. As shown in Fig. 7(f), if the optical receiving power of 4-km SMF and 3-m wireless transmitted OFDM data raises from −3 to 0 dBm, its BER can be optimized from 8.6 × 10^−3^ to 1.9 × 10^−3^ which falls below the FEC criterion and reveals a receiving power penalty of 1.1 dB. This guarantees the successful transmission of the MMW carried OFDM data; however, as the induced power fading is down-shifted into the preset modulation bandwidth of received OFDM data after 10-km SMF transmission, the degradation of SNR performance cannot be easily released by simply increasing the optical receiving power. Nevertheless, these results have declared the capability of the DMLD based fiber-wireless access architecture at 60 GHz for carrying the 16-QAM OFDM data at 6 Gb/s after 4-km SMF and 3-m wireless transmissions at sufficient optical transmitting power.

To further discuss the temperature related optical stability, the Fig. 7(g) shows the optical spectrum of the dual-mode injection-locked DMLD at different operating temperatures. Controlling the temperature at 22 °C optimizes the dual-mode injection-locking to exhibit a side-mode suppression ratio (SMSR) of 51.6 dB. When the operating temperature is decreased to 19 °C, the SMSR is slightly decreased to 51 dB because the intense mode competition plays a more important role than the enhanced quantum efficiency in the dual-mode injection-locked DMLD cavity, as shown in the left of Fig. 7(g). To avoid the water vapor condensed on the current package to damage the slave LD, the operating temperature of the slave DMLD at less than 19 °C was prohibited in this work. In addition, increasing the operating temperature up to 27 °C would also decreases the dual-mode injection-locking efficiency of the DMLD, which inevitably reduces the SMSR to 49.8 dB, as shown in the right of Fig. 7(g). Mode deviation occurs to further degrade the SMSR to 49.6 dB when continuously increasing the operating temperature beyond 28 °C. At different operating temperatures, the BtB transmission stability characterized by modulating the injection-locked DMLD with 16-QAM OFDM data at 6-Gb/s are discussed, and the receiving BERs measured are shown in Fig. 7(h). The lowest BER of 4.2 × 10^−4^ is obtained at an operating temperature of 22 °C, and the allowable operating temperature ranging between 19 °C and 27 °C is observed with corresponding BER met the demand of FEC criterion (below 3.8 × 10^−3^).

For the comparison with previously proposed schemes, although the optical carrier suppression (OCS) is a simple way to generate dual-mode optical carrier, the substantial suppression on central carrier is difficult and the residual central carrier would compete and share the transmission gain with the transmitted double-sideband carrier. This inevitably decreases the power budget, induces additional chromatic dispersion, attenuates the heterodyned MMW power, and degrades the transmitted baseband data. The advantage of our approach is to further suppress the OCS generated central carrier. With dual-mode injection-locking the DMLD, the central carrier can be further suppressed by at least 11.5 dB because of the inherent mode selecting mechanism of the DMLD cavity. Besides, typical OCS method requires either data encoder or frequency up-conversion mixer, which inevitably needs additional optical or MMW amplification to degrade the SNR and to raise system cost. In contrast, the slave DMLD used in this work simultaneously functions as the data encoder and the optical amplifier, which exhibits cost-effective and flexible features. In brief, the most distinguished part of this work as compared to others is that the DMLD with enhanced central carrier suppression can concurrently function as a data encoder and an optical amplifier to compact the whole system and save its cost with excluding the use of additional data modulator and frequency up-conversion mixer in typical OCS method. This is a novel scheme to be considered in addition to currently available MMWoF approaches.

## Conclusion

By simply using a directly modulated and IMD_3_ suppressed DMLD as an optical carrier for the MMW embedded fiber-wireless access, a 60-GHz MMWoF architecture is demonstrated for delivering the 6-Gb/s 16-QAM OFDM data over 4-km SMF and 3-m wireless transmissions. By enlarging the peak power of the dual-sideband master to 3 dBm, the throughput degradation of the DMLD can be mitigated with saturation effect, which simultaneously reduces its lasing threshold and RIN to 7.7 mA and −110.4 dBc/Hz, respectively. By examining the two-tone modulation analysis, the IMD_3_ spectral peaks can be suppressed to −85 dBm by implementing the dual-mode injection-locking at power of 3 dBm, which provides an improved SFDR of up to 85.8 dB/Hz^2/3^. This operation reduces the noise floor of the spectrum of the DMLD carried QPSK-OFDM data by 5 dB after optical BtB and MMW wireless transmissions. The waveform clipping and RIN induced intensity fluctuation can also be decreased at an injection power of 3 dBm, which reduces the corresponded PAPR to 13.5 dB at 10^−2^ probability and improves the SNR, EVM and BER of the QPSK-OFDM data to 16.5 dB, 14.9% and 8.5 × 10^−10^, respectively, after optical-BtB/3m-wireless transmission. By employing the 6-Gb/s 16-QAM OFDM data to increase the transmission capacity, the optimized optical receiving power at 0 dBm effectively restricts the power fading effect, which improves the BER of the 4-km SMF and 3-m free space transmitted 16-QAM OFDM data to 1.9 × 10^−3^ and reveals a receiving power penalty of only 1.1 dB.

## Methods

### The dual-sideband master injection-locked LD

**Figure 8:**
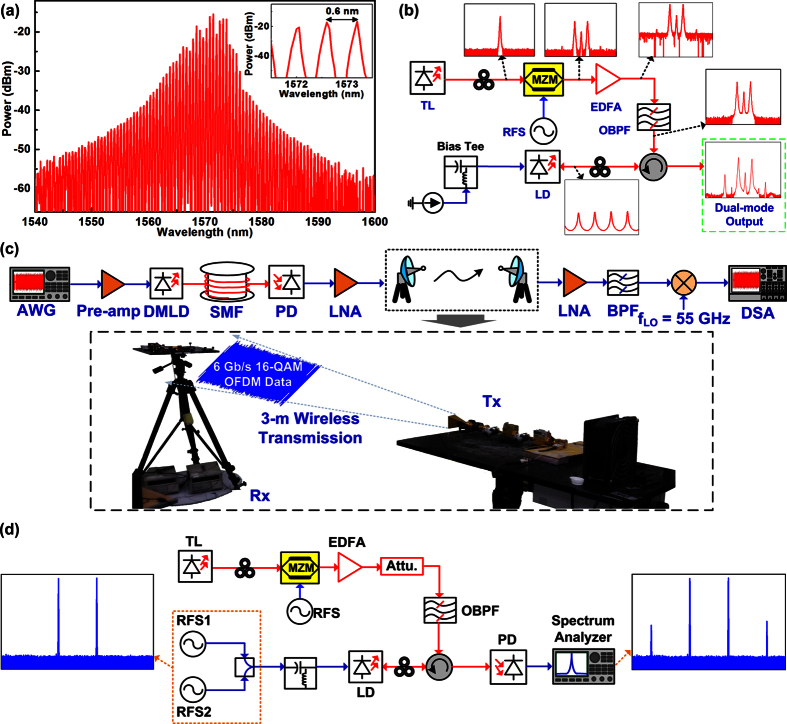
The experimental setup of the slave LD for dual-mode operation and further implementing a 60-GHz fiber-wireless access architecture. (**a**) The optical spectrum of the free-running slave LD. (**b**) The experimental setup of the slave LD injection-locked by a dual-sideband master. (**c**) The schematic diagram for the DMLD based 60-GHz fiber-wireless access architecture. (**d**) The testing bench for measuring the DMLD induced intermodulation distortion.

In experiments, a LD with a cavity length of 600 μm and a front-facet reflectance of 2% is employed as a slave light source, which reveals a lasing spectrum ranged between 1550 and 1590 nm with longitudinal mode-spacing of 0.6 nm, as shown in Fig. 8(a). As the mode spacing of the slave LD (Δ*f*_S_ = 75 GHz) is designed to be slightly larger than that of the master (Δ*f*_M_ = 60 GHz), the resonant FWM in the slave LD can be significantly suppressed by offsetting the injected mode from cavity resonance. By modulating the single-mode master (ANDO, AQ4321A) with a MZM that biased at null point and driven by a RF synthesizer (RFS, Agilent, E8257D) at 30 GHz, a central-carrier suppressed dual-sideband master light source with its dual-mode frequency spacing of 60 GHz can be synthesized for injection-locking the slave LD, as shown in Fig. 8(b). By optimizing the polarization of the dual-sideband master with a polarization controller (PC), the CCSR of the MZM generated dual-sideband master is optimized to 15.5 dB. After adding a commercial erbium-doped fiber amplifier (EDFA, Alnair Labs, LNA 200-C), the peak power of the dual-sideband master can be increased up to 3 dBm, and the residual amplified spontaneous emission was filtered by an optical band-pass filter (OBPF, Alnair Labs, TFF-15-1-SM-L-100-FS). Through an optical circulator and a PC, the dual-mode dual-sideband master was inserted into the slave LD for injection-locking its adjacent dual longitudinal modes so as to implement the dual-mode LD for downstream transmission (hereafter referred as DMLD).

### The DMLD based 60-GHz fiber-wireless access architecture

For wired and wireless transmissions, the schematic diagram for the DMLD based 60-GHz fiber-wireless access architecture is illustrated in Fig. 8(c). The DMLD is used for concurrently providing the downstream optical carrier to deliver the QAM-OFDM data and for remotely self-beating the 60-GHz MMW carrier, which is biased at 50 mA (2.5 times the threshold current of 20 mA) to offer a sufficient output power for the fiber wired baseband and MMW wireless transmission. To stabilize the output dual-mode wavelengths, the temperature of the DMLD was controlled at 22 °C by a homemade thermoelectric cooling module. The data to be delivered is a 3-Gb/s QPSK and a 6-Gb/s 16-QAM OFDM data, respectively, which encodes digital data onto 64 subcarriers within a bandwidth of 1.5 GHz. The electrical QAM-OFDM data is exported by an arbitrary waveform generator (AWG, Agilent, 849413) with a sampling rate of 12 GS/s, which directly modulates the DMLD after amplification. To enlarge the power of the electrical QAM-OFDM data, a linear pre-amplifier with small-signal gain of 26 dB was added after the output of the AWG. After passing through the SMF spools with lengths of 0, 4 and 10 km, respectively, the DMLD carried baseband QAM-OFDM data was received by a high-speed PD, which concurrently self-beats the dual-mode optical carrier with QAM-OFDM data into the 60-GHz MMW carrier. Such a MMW carrier with QAM-OFDM data is amplified by using a low-noise amplifier (LNA) and sent into a standard horn antenna for wireless transmission. At the wireless receiving end, the 3-m free-space transmitted QAM-OFDM data is received by another standard horn antenna and passed through a band-pass filter and a LNA, respectively, which is down-converted to an intermediate frequency at 5 GHz with a 55-GHz LO signal. After re-sampling by a digital serial analyzer (DSA, Agilent, 91604A) with 40-GS/s sampling rate, the received OFDM data is retrieved by a homemade MATLAB decoding program to analyze its BER, EVM and SNR performances.

### The testing bench for measuring the DMLD induced intermodulation distortion

To analyze the nonlinear modulation distortion induced in the DMLD under dual-mode injection, the two-tone diagnostic setup is shown in Fig. 8(d). By combining two outputs of RF synthesizers (RFSs, Anritsu MG3962C and Agilent, E4433B), the two-tone RF signal (ω_1_ and ω_2_) is employed to directly modulate the DMLD for observing its IMD_3_ terms (2ω_1_ − ω_2_ and 2ω_2_ − ω_1_) at low frequencies to conveniently survey the nonlinear modulation distortion. In particular, to match with the frequency division of the OFDM subcarriers, the frequency difference between the two-tone RF signals is set as 23.44 MHz. After transmitting through a SMF, the DMLD carried two-tone RF signal is received and converted by a PD (New Focus, 1434) before analyzing by a spectrum analyzer (HP, 8565E).

## Additional Information

**How to cite this article**: Tsai, C.-T. *et al*. 60-GHz Millimeter-wave Over Fiber with Directly Modulated Dual-mode Laser Diode. *Sci. Rep.* **6**, 27919; doi: 10.1038/srep27919 (2016).

## Figures and Tables

**Figure 1:**
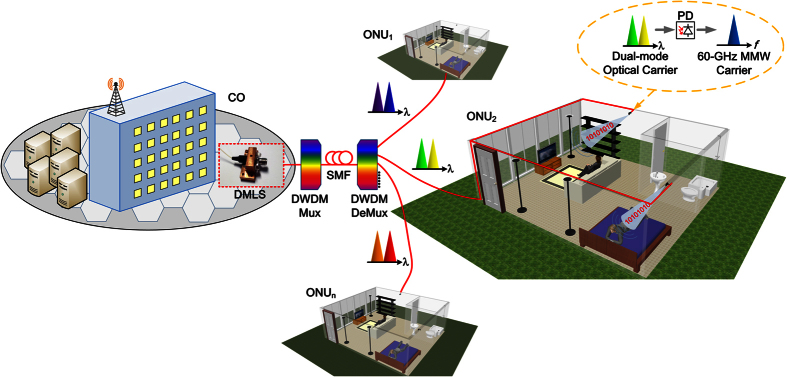
The DMLS based 60-GHz MMW embedded fiber-wireless access network. The DMLS based hybrid architecture for 60-GHz MMW fiber-wireless access can converge the current optical and wireless networks for indoor communications. CO: central office, DWDM Mux/DeMux: dense wavelength division multiplexing multiplexer/demultiplexer, ONU: optical network unit. *Sweet Home 3D, Copyright* (*c*) *2005–2016 Emmanuel PUYBARET/eTeks info@eteks.com.*
